# Single-cell transcriptomics systematically discloses the immune-inflammatory responses of peripheral blood mononuclear cells for diabetic nephropathy

**DOI:** 10.1016/j.gendis.2023.06.007

**Published:** 2023-07-13

**Authors:** Jiangpeng Wu, Nan Hu, Baochun Guo, Hualin Ma, Lulin Xie, Yisha Huang, Siyu Xia, Yuke Jiang, Zhijie Li, Jigang Wang, Xinzhou Zhang, Zhen Liang

**Affiliations:** a Department of Nephrology, Shenzhen Key Laboratory of Kidney Diseases, and Shenzhen Clinical Research Centre for Geriatrics, Shenzhen People's Hospital (The Second Clinical Medical College, Jinan University, The First Affiliated Hospital, Southern University of Science and Technology), Shenzhen, Guangdong 518020, China; b Department of Geriatrics, and Shenzhen Clinical Research Centre for Geriatrics, Shenzhen People's Hospital (The Second Clinical Medical College, Jinan University, The First Affiliated Hospital, Southern University of Science and Technology), Shenzhen, Guangdong 518020, China; c State Key Laboratory for Quality Ensurance and Sustainable Use of Dao-di Herbs, Artemisinin Research Center, and Institute of Chinese Materia Medica, China Academy of Chinese Medical Sciences, Beijing 100700, China

Diabetic nephropathy (DN) has become the leading cause of end-stage renal disease with high morbidity and mortality among individuals with diabetes mellitus. Although functional alterations of renal infiltrating immune cells have been reported as part of the pathological mechanism of DN, the understanding of the immune response underlying peripheral blood mononuclear cells (PBMCs) in DN remains limited. Here, single-cell RNA sequencing (scRNA-seq) was used to profile the transcriptomic signatures of PBMCs from DN patients. We identified four well-known cell types of PBMCs and analyzed their respective cell subtypes. The underlying biological processes were captured from the altered cell (sub)types. A number of cell-type-specific cytokines and transcription factors driving the DN-associated transcriptomic changes were detected. Taken together, our data revealed detailed and rich single-cell transcriptomic signatures of PBMCs from DN patients and identified cell-type-specific pathways and molecules associated with the mechanisms of DN progression.

To characterize the transcriptomic profiling and cellular composition of DN patients, PBMC samples from three patients and four healthy controls (HC) were collected for scRNA-seq. The entire research process was briefly summarized in Figure 1A. After strict quality control, a total of 60,903 cells (29,218 for DN and 31,685 for HC) were obtained for subsequent analysis (Fig. S1A). These cells were integrated and divided into four different subsets, including T/natural killer (NK) cells, myeloid cells, B cells, and platelets (Fig. 1B; Fig. S1B). They were annotated by canonical marker genes (Fig. 1C and Table S3) and specific functional enrichment (Fig. S1C). The major cell types were T/NK cells (40,333, 66.22%) and myeloid cells (15,149, 24.87%) (Fig. 1D and Table S4). We then compared the changes in the proportions of the four cell types between healthy controls and DN patients. Myeloid cells were significantly increased, whereas B cells were markedly decreased in the DN group (Fig. 1D). No dramatic changes were observed in T/NK cells and platelets in the two groups.

**Fig. 1:**
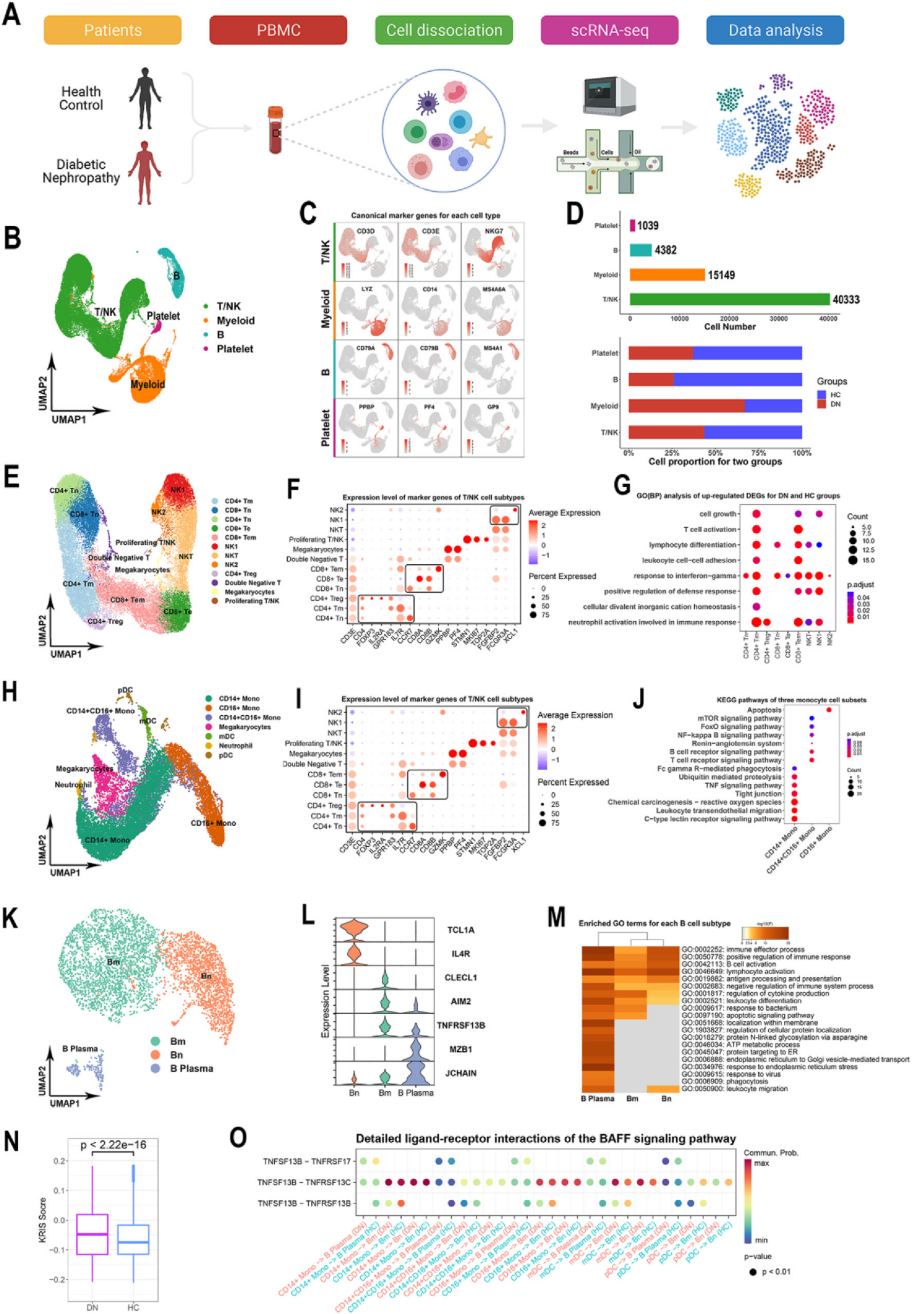
The single-cell transcriptomic signatures of PBMCs from DN patients and healthy controls. **(A)** An overview of the study workflow. **(B)** UMAP plot for four immune cell types. **(C)** The expression of canonical marker genes for four immune cell types. **(D)** The histogram of cell numbers for four immune cell types (top) and the proportion changes of four immune cell types for DN and HC groups (bottom). **(E)** UMAP plot for T/NK cell subtypes. **(F)** The dot plots showing the expression of canonical marker genes identified T/NK cell subtypes. **(G)** GO analysis for up-regulated DEGs of different T/NK cell subtypes. **(H)** UMAP plot for myeloid cell subtypes. **(I)** The dot plots showing the expression of canonical marker genes identified myeloid cell subtypes. **(J)** The bubble plot for specific KEGG pathways of three monocyte cell subsets. **(K)** UMAP plot for B cell subtypes. **(L)** Violin plots showing the expression of canonical marker genes identified B cell subtypes. **(M)** Heatmap of enriched GO terms for each B cell subtype. **(N)** KRIS score was composed of 17 special genes between the DN and HC groups. **(O)** Detailed ligand–receptor interactions of the BAFF signaling pathway. UMAP, uniform manifold approximation and projection; GO, Gene Ontology.

To investigate the potential role of T/NK cells in DN development, we re-clustered these cells at the appropriate resolution and identified 12 cell subsets using canonical marker genes (Fig. 1E, F; Fig. S2A). CD4^+^ T naïve (Tn) and CD8^+^ Tn cells were greatly decreased in the DN group, which may indicate T cell exhaustion in DN patients (Fig. S2B, C and Table S6). We found the differentially expressed genes (DEGs) and functional transformations (Fig. 1G) for each T/NK cell between the two groups. All cell subtypes were involved in the response to interferon-gamma except CD4^+^ regulatory T (Treg) cells, suggesting the existence of a widespread proinflammatory state in the circulation of DN patients. This state could promote the recruitment of more inflammatory cells to the kidney and advance the development of DN. Taken together, the current data revealed that T cells and NK cells showed an activated proinflammatory state, which could be an important factor contributing to renal impairment in DN.

Myeloid cells exhibited a significant alteration in the DN group, suggesting a dramatic immune response. The myeloid cells were re-clustered to identify more sophisticated transcriptional features (Fig. 1H, I). Monocytes were divided into three typical clusters, namely, CD14^+^ monocytes (Mono), CD16^+^ Mono, and CD14^+^ CD16^+^ Mono (Fig. S3A). The remaining clusters were annotated for megakaryocytes, myeloid dendritic cells (mDC), plasmacytoid dendritic cells (pDC), and neutrophils (Fig. S3A). CD14^+^ monocytes were markedly decreased while CD14^+^ CD16^+^ monocytes were significantly increased in the DN group (Fig. S3B and Table S10). Subsequently, all DEGs of monocyte subtypes were subjected to Kyoto Encyclopedia of Genes and Genomes (KEGG) analysis (Fig. 1J). CD14^+^ Mono cells were specifically enriched in the reactive oxygen species (ROS) and tumor necrosis factor (TNF) signaling pathways compared to the HC group. ROS production has been proven to cause irreversible kidney injury through various mediators.^1^ The TNF pathway could be activated in monocytes from DN patients and correlated with the activation of nuclear factor-kappa B (NF-κB) signaling.^2^ CD14^+^ CD16^+^ Mono cells were involved in the T/B cell receptor signaling pathway and the NF-κB signaling pathway, indicating the existence of extensive communication between different cell (sub)types. Only the apoptosis pathway of CD16^+^ Mono cells in the DN group was significantly different from that of the HC group. Oxidative stress-induced apoptosis was frequently detected in CD16^+^ Mono cells. Collectively, our scRNA-seq data on myeloid cells revealed an activated and pro-inflammatory status of monocytes in DN patients.

Compared with T/NK cells and myeloid cells, the role of B cells in the development of DN has been less studied. Some studies suggest that B cells may secrete various proinflammatory cytokines, regulate T-cell function, and induce the formation of immune complexes.^3^ In our study, B cells experienced a dramatic decrease from a healthy state to DN disease (Fig. 1D; Fig. S4A). B cells were re-clustered to further investigate the changes in cell subtypes. According to the canonical marker genes, B cells were composed of three subtypes including B naïve cells (Bn), B memory cells (Bm), and B plasma cells (Fig. 1K, L). Functional enrichment analysis demonstrated that these subtypes were widely involved in B cell activation, immune effector process, regulation of cytokine production, and antigen processing and presentation (Fig. 1M).

Numerous evidences have revealed that cytokines are not only extensively involved in the regulation of immune responses, but also play important pleiotropic roles in kidney injury.^4^ We investigated the expression profile of cytokines between two groups. Chemotactic cytokines (*CCL3*, *CCL4*, and *CX3CR1*) and TNF superfamily members (*TNFSF10*, *TNFSF13B*, *TNFRSF1B*, and *TNFRSF1A*) were highly expressed, suggesting that they may play a potential role in the pathogenesis of DN (Fig. S5A). The research by Niewczas et al. identified a kidney risk inflammatory signature (KRIS) comprising 17 circulating inflammatory proteins and confirmed that several TNF receptor superfamily (TNF-RSF) members were robustly associated with the progressive renal decline, especially *TNFRSF1A* and *TNFRSF1B*.^5^ All of the genes corresponding to the 17 circulating proteins in the KRIS were identified in our data (Fig. S5B). We then analyzed the total expression of the 17 genes constituting the KRIS score and confirmed that the score was significantly higher in the DN group (Fig. 1N).

To unravel the potential intercellular signaling patterns hidden behind the DN process, the CellChat program was used to infer statistically and biologically significant communication networks. By comparing the communication features, the interaction strengths of both groups were mainly concentrated on B cells interacting with the monocytes and DC cells (Fig. S6A). Interestingly, *CXCL*, *IFN-II*, and *OX40* signaling pathways were present only in the DN group, whereas *BTLA*, *CD40*, and *IL1* signaling pathways were present only in the HC group (Fig. S6B, C). More importantly, many members of the TNF superfamily were widely involved in cell–cell communication. The most prominent signaling pattern was the B-cell-activating factor of the TNF family (BAFF, also known as tumor necrosis factor ligand superfamily member 13B) pathway including *TNFSF13B*-*TNFRSF17*, *TNFSF13B*-*TNFRSF13C*, and *TNFSF13B*-*TNFRSF13B* (Fig. 1O), which was consistent with the finding that *TNFSF10* and *TNFSF13B* genes of TNF-RSF members were up-regulated in the monocytes of the DN group. The BAFF pathway ligands were mainly expressed in monocyte subtypes, mDC and pDC, while the receptors were mainly concentrated in B cell subtypes, indicating that B cells were widely involved in the pathogenesis of DN (Fig. S6D).

In conclusion, our study revealed the transcriptomic signature of PBMCs from DN patients and healthy controls at single-cell resolution. We identified a number of cell-type-specific pathways and molecules that drive the DN-associated transcriptomic changes. These findings may provide insights into the underlying immune responses during DN progression and improve the understanding of DN pathogenesis.

## Ethics declaration

All study procedures were approved by the Institutional Review Board of the Shenzhen People's Hospital and conducted in accordance with the Declaration of Helsinki. Each participant was informed of the purpose of the study and signed the written informed consent.

## Author contributions

Jiangpeng Wu, Nan Hu, Baochun Guo, and Hualin Ma performed the data analysis and wrote the manuscript. Lulin Xie conducted the PBMC isolation, library construction for sequencing, and data collection. Yisha Huang managed the samples. Siyu Xia and Yuke Jiang carried out preliminary data analysis. Zhijie Li, Jigang Wang, Xinzhou Zhang, and Zhen Liang designed the whole study, supervised all aspects of the study implementation, interpreted the data, and contributed to the revision and editing of the manuscript.

## Conflict of interests

The authors declare no conflict of interests.

## Funding

The current study was supported by the Shenzhen Fund for Guangdong Provincial High-level Clinical Key Specialties (China) (No. SZGSP001), Shenzhen Governmental Sustainable Development Fund (Guangdong, China) (No. KCXFZ20201221173612034), the National Natural Science Foundation of China (China) (No. 82170842), the Natural Science Foundation of Shenzhen City (China) (No. KCXFZ20201221173600001), Guangdong Basic and Applied Basic Research Foundation (China) (No. 2021A1515012164), and Shenzhen Key Laboratory of Kidney Diseases (No. ZDSYS201504301616234).
