# Supplementary material for: Single-cell transcriptomics systematically discloses the immune-inflammatory responses of peripheral blood mononuclear cells for diabetic nephropathy
Source: Genes Dis. 2023 Jul 13;11(4):101033. doi: 10.1016/j.gendis.2023.06.007 (PMC10951445; doi:10.1016/j.gendis.2023.06.007)
Supplement: Multimedia component 2 [file mmc2.docx]

**Single-cell transcriptomics systematically discloses the immune inflammatory responses of peripheral blood mononuclear cells for diabetic nephropathy**

**1. Important Materials and Methods**

**1.1 Patients**

Whole blood samples were collected from 3 DN patients (one male and two females) and 4 healthy controls (three males and one female) admitted to the Department of Nephrology, Shenzhen People’s Hospital. The diagnosis of the DN patients depended on the combination of clinical symptoms, past medical history and laboratory tests by at least two experienced clinicians. They all suffered from hyperglycemia for more than 10 years and severe hypertension. Four healthy donors were recruited from routine physical examinations and had no history of hyperglycemia, hypertension and hyperlipidemia. The characteristics of these participants are summarized in **Table S1**. All study procedures were approved by the Institutional Review Board of the Shenzhen People’s Hospital and conducted in accordance with the Declaration of Helsinki. Each participant was informed of the purpose of the study and signed the written informed consent.

**1.2 PBMC isolation**

The PBMCs were isolated from venous blood using a Ficoll-Hypaque density solution according to standard density gradient centrifugation methods. After centrifugation, the cells were washed twice with PBS and resuspended in PBS with 0.04% BSA. Cell viability was greater than 90% for each sample.

**1.3 Single-cell preparation and sequencing**

Single-cell suspensions of the scRNA-seq samples were harvested and barcoded using the Chromium Next GEM Single Cell 3’ Kit v3.1 (10x Genomics, 1000268), Chromium Next GEM Single Cell 3’ Gel Bead Kit v3.1 (10x Genomics, 1000122) and Chromium Next GEM Chip G Single Cell Kit (10x Genomics, 1000120). Single cell libraries were constructed using the Library Construction Kit (10x Genomics, 1000196) according to the manufacturer’s instructions.

**1.4 scRNA-seq data analysis**

Raw single-cell sequencing data were processed using the Cell Ranger pipeline (version 6.0.1) with recommended parameters and aligned to the human reference genome (GRCh38). 6828-11567 (average 9291) cells were acquired for individual libraries and 916-2066 (average 1382) genes were identified with UMIs per cell. The gene-barcode matrix containing barcoded cells and gene expression counts was loaded into the Seurat (version 4.0.3) R toolkit for further analysis. The possible low quality or double nucleated cells were excluded by the following criteria with nFeature_RNA from 200 to 4000 and percentage of mitochondrial genes < 15%. The detailed information of the filtering parameters and cell counts were listed in **Table S2**. The datasets for all samples were individually log-normalized and the top 2000 highly variable genes were detected. All sample datasets were then integrated with the identified anchors and the integrated matrix was scaled to correct technical differences between samples. The top 30 dimensions were obtained by performing principal component analysis (PCA), and cluster analysis was conducted based on shared nearest neighbor graphs to identify cell clusters. Meanwhile, the resolutions were set to 0.05 for the major cell types of PBMCs, 0.6 for T/NK cells, 0.2 for myeloid cells and 0.1 for B cells. Cell subtypes were visualized using Uniform Manifold Approximation and Projection (UMAP), and the cell labels were annotated based on canonical marker genes.

**1.5 Differential expression and functional enrichment analysis**

Differential expression genes (DEGs) analysis for each cell type was performed with “FindMarkers” function in Seurat packages with adjusted logfc.threshold = 0.1. Significant DEGs were defined by the two parameters p < 0.05 and |log2FC| > 0.1. Pathway enrichment analysis was performed using GO analysis, KEGG analysis, GSEA, GSVA and Metascape web tools ([https://metascape.org/gp/index.html#/main/step1](https://metascape.org/gp/index.html%23/main/step1)) according to different requirements. GO and KEGG analysis were performed by clusterProfiler package for significant DEGs. GSEA was implemented using fgsea package for all DEGs of four major immune cell types and GSVA was executed by GSVA R package for significant DEGs of T/NK cells between groups. P < 0.05 was considered as significantly enriched for all functional enrichment analyses.

**1.6 Cell-cell communication**

Cell-cell communication analysis was performed by the R CellChat package with default parameters. Two CellChat objects were generated with single-cell data from the DN and HC groups. The two objects were merged via the “mergeCellChat” function for comparative analysis. Cell type labels were derived from all subsets of T/NK cells, myeloid cells, B cells and platelets except megakaryocytes. We inferred the intercellular communication between all cell subsets of the two groups and showed some specific signaling pathways by chord diagram. The results of the cell-cell communication analysis were then combined with the differential gene expression to prioritize the interactions most likely to be associated (based on the association) with DN pathological mechanisms. The curated human CellChat database is greatly resourceful in potential signaling ligand-receptor pairs collected from the literature.

**1.7 Transcription factor analysis**

Transcription factor analysis was performed using Single-Cell Regulatory Network Inference and Clustering (SCENIC) which contribute to inferring transcription factors for deciphering gene regulation. First, GENIE3 was used to infer TF gene co-expression modules from single-cell sequencing data. Then, TF motif enrichment analysis was performed using RcisTarget to obtain effective regulons containing TFs and their putative direct-binding targets. Next, the activity of each regulon for all cells was calculated using AUCell project by assessing whether target genes were enriched in the top quantile of the cell signature. Finally, we calculated to obtain the heatmap of TF regulation intensity for subsets of T/NK cells, myeloid and B cells.

**2. Additional Results**

**2.1 Four major immune cell types are identified in the blood**

After single-cell preparation of PBMCs, approximately 20000 cells per sample were loaded onto the 10× Genomics platform for scRNA-seq. And approximately 9000 cells per sample were recovered from the sequencing data via the standard Cell Ranger protocol. The detailed filtration parameters and the specific sample information were listed in the **Fig. S1A** (**Table S2**). The four immune cell types were visualized by UMAP analysis. GSEA showed that the DN group exhibited several significantly up-regulated pathways compared to the HC group including interferon gamma/alpha response, TNFA signaling via NF-κB, apoptosis, inflammation response and IL6 JAK STAT3 signaling (**Fig. S1D**). All of them have been demonstrated the importance in the progression of DN.[^1-4^](#_ENREF_1) T/NK and myeloid cells may be primarily responsible for these regulatory responses because of changes in the pathways and proportions. Interestingly, the data displayed that T/NK and myeloid cells were highly involved in the interferon alpha/gamma response (**Fig. S1E**, **Table S5**). It suggested important effects of interferon response in the DN progression, which is consistent with previous reports about DN studies.[^5^](#_ENREF_5)^,^ [^6^](#_ENREF_6)

**2.2 Activated T cells and NK cells show proinflammatory state in DN blood**

T cells have been extensively studied in the pathogenesis of diabetes and DN. In the hyperglycemic milieu, T cells could remain the activated state through multiple pathways, leading to kidney damage such as albuminuria and fibrosis.[^7^](#_ENREF_7) Twelve cell subtypes were visualized by UMAP plot. The major cell types consisted of CD4+ T cells (CD3E, CD4), CD8+ T cells (CD3E, CD8A, CD8B), NKT (CD3E, FGFBP2, FCGR3A), NK1 cells (FGFBP2, FCGR3A) and NK2 cells (GZMK, XCL1). And there were small numbers of double negative T cells, megakaryocytes (PPBP, PF4) and proliferating T/NK cells (STMN1, MKI67, TOP2A). Specifically, CD4+ T cells were composed of CD4+ Tn cells (CCR7), CD4+ Tm cells (GPR183, IL7R) and CD4+ Treg cells (FOXP3, IL2RA). CD8+ T cells were subdivided into CD8+ Tn cells (CCR7), CD8+ Te (ZNF683) and CD8+ Tem (GZMK). Given the critical role of T and NK cells in the progression of DN, the main focus was to investigate the changes in the proportion of each T/NK cell subtype in the two groups. NK1 cells were the dominant proportion of NK cells and were responsible for phagocytosis and the NF-κB signaling pathway. NK2 cells were involved in lymphocyte activation and constituted a minor part of NK cells. The increase of NK cells mellitus has been previously reported in type 2 diabetes, although further studies were needed to clarify the functions of NK cells in the development of DN.[^8^](#_ENREF_8) The up-regulated DEGs of three significantly altered cell types (CD4+ Tn, CD8+ Tn and NK2) were used for functional enrichment analysis (**Fig. S2F, Table S7, 8, 9**). Functional enrichment was then performed by gene set variation analysis (GSVA) based on the total DEGs of T/NK cells between DN and HC groups. There were several up-regulated pathways associated with DN pathogenesis including type II diabetes mellitus, arachidonic acid metabolism, aldosterone regulated sodium reabsorption, natural killer cell mediated cytotoxicity and apoptosis as shown in **Fig. S2G**, which is highly consistent with the DN scenario, thereby suggesting that the scRNA-seq is an authentic technology to elucidate disease mechanisms and profile the global landscape of immune cells in the blood. Finally, some key TFs were identified for each cell type (**Fig. S2H**). For example, FOXP1 shows significantly lower expression in CD4+ Tn cells of the DN group (**Fig. S2H**). It has been shown that FOXP1 could prevent high glucose-induced proliferation, oxidative stress, and extracellular matrix accumulation in renal mesangial cells of the kidney,[^9^](#_ENREF_9)^,^ [^10^](#_ENREF_10) suggesting that suppression of FOXP1 might exacerbate the progression of DN.

**2.3 Monocytes in DN patients are widely activated in the proinflammatory pathways**

Monocytes or macrophages are the major inflammatory immune cells that can produce various mediators to accelerate the progression of DN; evidence suggests that monocytes are highly involved in the pathogenesis of glomerulopathy and tubulointerstitial lesion in DN.[^11^](#_ENREF_11) GO analysis for the overall up-regulated DEGs of myeloid cells revealed several activated pathways involved in response to interferon-gamma/type I interferon, positive regulation of cytokine production/cell adhesion and response to tumor necrosis factor/oxidative stress (**Fig. S3C**). These inflammatory and cytokine-related pathways are closely related to the development of DN and also imply the activated status of myeloid cells in DN patients.[^12-15^](#_ENREF_12). Moreover, the most interesting finding was the altered proportion of each monocyte subtype. The similar pattern shifts of these three monocyte subtypes have been identified by previous studies with conventional research methods.[^16^](#_ENREF_16)^,^ [^17^](#_ENREF_17) The increase of CD14+CD16+ monocytes has been demonstrated in DN patients and it is highly associated with diabetic severity and higher rates of cardiovascular events and death in diabetic patients.[^11^](#_ENREF_11)^,^ [^18^](#_ENREF_18)^,^ [^19^](#_ENREF_19) The number of DEGs between the DN and HC groups also laterally reflected the immune response to the disease. CD14+ Mono, CD16+ Mono and CD14+CD16+ Mono cells had the largest number of unique DEGs (**Table S11, 12, 13**). And the unique DEGs of three monocyte subtypes were subjected to functional enrichment analysis (**Fig. S3D, E, F**). Finally, several key TFs were revealed in each monocyte type (**Fig. S3G**). Interestingly, the transcription factor 7 (TCF7) gene was mainly expressed in CD14+CD16+ monocytes and had relatively lower expression in DN group than in the HC group (**Fig. S3H**). To the best of our knowledge, the role of TCF7 in the progression of DN remains unexplored. One study indirectly implicated that TCF7 might function as a target of miR-22-3p which could upregulate gluconeogenesis in the pathogenesis of diabetes.[^20^](#_ENREF_20)

**2.4 B cell subsets are highly involved in the DN pathogenesis**

Although the proportion of B cells for DN patients was significantly lower in DN patients than in healthy controls, no significant changes were observed in the proportion of each subtype from healthy state to diseased state (**Fig. S4A, B, C, Table S14**). Finally, TFs were also analyzed and assessed. Only four TFs came out and all of them might have a crucial influence on the Bm cell subtype (**Fig. S4D, E**). Among the four factors, Krüppel-like factor 2 (KLF2) has been demonstrated that it can regulate the proliferation, differentiation, and trafficking of B cells.[^21^](#_ENREF_21) And upstream stimulatory factor 2 (USF2) has been shown to enhance the glucose-induced expression of TGF-β1 in mesangial cells and accelerate the kidney injury in the progression of DN possibly through the deposition of extracellular matrix components.[^22^](#_ENREF_22)^,^ [^23^](#_ENREF_23)

**2.5 Cytokines of TNF superfamily are the pivotal factors in the blood of DN**

A growing understanding of the complex interactions between pro-inflammatory cytokines and the pathogenesis of DN has demonstrated that cytokines are strongly associated with the development and progression of the microvascular diabetic complications.[^24^](#_ENREF_24) We investigated the expression profile of cytokines on diabetic kidney injury from various aspects including two groups and various cell subtypes to accurately identify new potential targets and develop innovative anti-inflammatory treatment strategies. First, all differentially expressed cytokines from DN and HC groups were listed in **Fig. S5A**. For example, CCL4 was generally considered to be a typical inflammatory factor associated with microvascular complications. The highlighted factors in the table were significantly up-regulated in the DN group, and the rest of the factors only showed an apparent up-regulation trend, which might be due to the limited number (n=3-4) in this study (**Fig. S5B**). Subsequently, we further analyzed the overall expression of 17 genes constituting the KRIS score, which definitely implicated that scRNA-seq technology is a powerful tool to systematically identify the biomarkers and disclose the mechanisms of disease development and progression, and could be mutually used with conventional study strategies to verify the results. Our scRNA-seq data suggested that chemotactic cytokines were mainly concentrated on T/NK cell subsets (**Fig. S5C**). Higher levels of CCL4 and CCL3 in the DN group were mainly distributed in NK cells and CD8+ T cells. CCL5, CXCR4, CCL4L2 and IL10RA were present only in T/NK cell subsets (of the DN group) but they did not differ in overall levels between two groups. Meanwhile, the TNFSF10 and TNFSF13B in the monocyte subsets were significantly higher in the DN group (**Fig. S5D**). IL3RA1 and IL1B, the signature genes for CD14+ Mono cluster, were highly expressed in the DN group but not in the HC group. Taken together, the significant advances from scRNA-seq have led to a better understanding of the relationship between the identified inflammatory cytokines and life-threatening diabetic nephropathy, and are useful to reveal important information on disease pathogenesis and to develop new treatment options for further clinical practice.

**2.6 B cells intimately interacts with monocytes through BAFF pathway in the DN blood**

The above data indicate that there are significant and differential changes on the immune cell landscape as an individual transitions from healthy to DN condition. Not surprisingly, these changes presented by DN disease could lead to abundant changes in the intricate cell-cell communication networks among these immune cells. Comparing the detailed signaling communication characteristics of the two groups, the number and strength of interactions in the DN group were slightly weaker than those in the HC group (**Fig. S7A, B**). Both up-regulated receptor-ligand pairs and down-regulated receptor-ligand pairs are illustrated in the DN group (**Fig. S7C**).

**3. Discussion on additional results**

In recent years, increasing evidence has highlighted the notion of inflammatory factors as contributors to the cardinal pathogenetic mechanisms of DN.[^25^](#_ENREF_25)^,^ [^26^](#_ENREF_26) Most studies on DN inflammation focus on local renal tissue and infiltrating immune cells. Few studies disclose the systemic inflammatory responses during DN progression.[^27^](#_ENREF_27) The purpose of this work is to elucidate the impact of DN disease on immune cell subsets and cytokine regulatory responses under high glucose conditions in the blood circulation. In particular, single-cell technology allows the analysis of all immune cell subtypes simultaneously with unprecedented resolution and provides more meaningful insights into the changes of systemic inflammatory factors in the DN process.[^28^](#_ENREF_28) A total of over 60,000 cells were obtained from 7 PBMC by strict filtration parameters and identified as four representative types (T/NK cells, myeloid cells, B cells and platelets) by canonical marker genes and functional enrichment. The number of T/NK cells showed little change in the DN and HC groups, while myeloid cells were greatly increased and B cells were dramatically reduced compared with the HC group. It is widely accepted that there are abnormal B cells and myeloid cells in diabetes mellitus, which are involved in the progression of DN from previous studies.[^29^](#_ENREF_29)^,^ [^30^](#_ENREF_30) In addition, T/NK cells and myeloid cells in the DN group strongly up-regulated interferon alpha/gamma response. Interferon gamma is regarded a pro-inflammatory cytokine in both T/NK cells and myeloid cells to accelerate the immune responses that are responsible for diabetic kidney injury reported in a large number of studies.[^31^](#_ENREF_31)^,^ [^32^](#_ENREF_32)

Re-clustering analysis revealed significant decrease in CD4+ Tn and CD8+ Tn cell populations in the DN group. The underlying mechanism of the change in naive T cells is that they may differentiate into functional effector cells or perform upregulated apoptotic function.[^33^](#_ENREF_33) Inferring analysis of TFs also confirmed that the low expression of FOXP1 has the ability to enhance the differentiation and apoptosis of naive T cells.[^34^](#_ENREF_34) It is an interesting finding that NK2 cells with highly expressed GZMK and XCL1 genes are significantly increased in the DN group, suggesting that it may be a meaningful cell subset for diagnosis and treatment of DN, which to our knowledge has not been reported by previous studies. Another important finding is that almost all T/NK cell subsets are involved in pathways associated with DN pathogenesis, indicating that T/NK cells in the DN blood maintain an activated state. Previous reports have confirmed that overactivation of the immune response can trigger further inflammation and oxidative stress in the diabetic kidney.[^1^](#_ENREF_1)^,^ [^6^](#_ENREF_6) We also identified another group of TFs including FOS and its heterodimeric binding partners JUN, JUNB and JUND. They may have direct responses to the activation of CD4+ Tm and CD8+ Tem cells. The TF genes can form the AP-1 complex causing increased fibronectin expression and extracellular matrix (ECM) synthesis to exacerbate DN progression upon T cell activation.[^35^](#_ENREF_35)^,^ [^36^](#_ENREF_36) The most interesting changes in myeloid cells are the monocyte-associated subsets in the DN group, especially the decreased CD14+ Mono and increased CD14+CD16+ Mono. It is well known that CD14+ monocytes are capable of phagocytosis, innate sensing/immune response and migration, while CD14+CD16+ monocytes function through antigen presentation, cytokine secretion, apoptosis regulation and differentiation.[^18^](#_ENREF_18) The pro-inflammatory CD14+CD16+ Mono may be involved in the microinflammation of DN through the pathway of TLRs/NF-κB/STAT as reported in a previous study.[^11^](#_ENREF_11) The specific KEGG results also indicate that CD14+CD16+ Mono in the DN group have significantly up-regulated NF-κB signaling pathway and T/B cell receptor signaling pathway. However, some studies on CD14+ Mono from PBMCs are contradictory, which may be due to the variation in disease progression.[^37^](#_ENREF_37) TCF7, an important TF for CD14+CD16+ Mono, responds to the Wnt signaling pathway by binding to the coactivator β-catenin to regulate downstream target genes, involved in cell proliferation, differentiation and growth processes.[^20^](#_ENREF_20) CEBPB and SPI1 are critical TFs in CD16+ Mono and essential for the expression of IL1B for inflammatory and immunoregulatory function as confirmed by previous literature.[^38^](#_ENREF_38)^,^ [^39^](#_ENREF_39)

Inflammatory mediators are important elements in the progression of DN. It was evident that highly expressed factors in the DN group were chemokines mainly found in T/NK cell subsets and members of the TNF superfamily mainly distributed in monocyte subsets. A number of studies have indicated that these inflammatory mediators have a persistent potency for the disease progression.[^40^](#_ENREF_40)^,^ [^41^](#_ENREF_41) Recent evidence has unraveled that CCL3 and CCL4 have chemoattractive ability to various immune cells, thus promoting a variety of pro-inflammatory processes in diabetes mellitus.[^42^](#_ENREF_42) Our results showed that higher levels of CCL3 and CCL4 in the DN group were mainly expressed in NK cells and CD8+ T cells. In addition, cell-cell interaction analysis also revealed that CCL and CXCL signaling was mainly detected in T cells, NK cells and monocytes. CX3CR1 is an important chemokine receptor that was highly expressed in CD8+ Tem and CD16+ Mono cells compared to the HC group, which is consistent with the published reports.[^43^](#_ENREF_43)^,^ [^44^](#_ENREF_44) It may be activated in inflammatory kidney tissue by high glucose levels and other cytokines (interferon-γ and TGFβ1).[^45^](#_ENREF_45) In addition to chemokines, members of the TNF superfamily are of particular interest in this study. Niewczas et al. constructed the KRIS consisting of 17 highly expressed circulating proteins that were enriched for members of the TNF receptor superfamily, which was strongly associated with the risk of end-stage renal disease in diabetes.[^46^](#_ENREF_46) All of the corresponding genes of the 17 proteins were also identified from our transcriptome data, including TNFRSF1A and TNFRSF1B, and they are significantly higher in the DN group than the HC group. Our scRNA-seq data are highly consistent with the reported study, indicating that the scRNA-seq method is reliable and accurate, and also implying that our findings in this paper are solid and convincing. Growing evidence supports the notion that elevated TNFRSF1A and TNFRSF1B may contribute to the progression of diabetic kidney disease.[^47^](#_ENREF_47) In this study, we found that TNFSF10 was highly expressed only in the CD14+ Mono and CD16+ Mono cells of the DN group. And many related studies have confirmed that the increased expression of TNFSF10 can aggravate renal injury by inducing apoptosis of tubular cells.[^48^](#_ENREF_48)^,^ [^49^](#_ENREF_49) Another interesting finding is that the expression level of TNFSF13B (BAFF) for the DN group is higher in CD16+ Mono cells. Moreover, cell-cell interaction analysis showed that BAFF signaling mainly occurs between B and myeloid cell subsets, and involves TNFSF13B-TNFRSF17, TNFSF13B-TNFRSF13C and TNFSF13B-TNFRSF13B interactions, suggesting the essential role of B cells in DN pathogenesis.

**4. Availability of data and material**

The raw scRNA-seq datasets used during the current study are available from the corresponding author on reasonable request.

**References**

1. F Imani, Y Horii, M Suthanthiran, et al. Advanced glycosylation endproduct-specific receptors on human and rat T-lymphocytes mediate synthesis of interferon gamma: role in tissue remodeling J Exp Med, 178(6)(1993), pp. 2165-2172.

2. H Ha, MR Yu, YJ Choi, et al. Role of high glucose-induced nuclear factor-κB activation in monocyte chemoattractant protein-1 expression by mesangial cells J Am Soc Nephrol, 13(4)(2002), pp. 894-902.

3. S Sifuentes-Franco, DE Padilla-Tejeda, S Carrillo-Ibarra, et al. Oxidative stress, apoptosis, and mitochondrial function in diabetic nephropathy Int J Endocrinol, 20182018), pp. 1875870.

4. E Feigerlová, S-F Battaglia-Hsu. IL-6 signaling in diabetic nephropathy: from pathophysiology to therapeutic perspectives Cytokine Growth Factor Rev, 372017), pp. 57-65.

5. B-Y Li, W Tan, J-L Zou, et al. Role of interferons in diabetic retinopathy World J Diabetes, 12(7)(2021), pp. 939-953.

6. C-C Wu, H-K Sytwu, K-C Lu, et al. Role of T cells in type 2 diabetic nephropathy Exp Diabetes Res, 20112011), pp. 514738.

7. L Kong, S Andrikopoulos, RJ MacIsaac, et al. Role of the adaptive immune system in diabetic kidney disease J Diabetes Investig, 13(2)(2022), pp. 213-226.

8. V Mxinwa, PV Dludla, TM Nyambuya, et al. Natural killer cell levels in adults living with type 2 diabetes: a systematic review and meta-analysis of clinical studies BMC Immunol, 21(1)(2020), pp. 51.

9. X Feng, H Wang, H Takata, et al. Transcription factor Foxp1 exerts essential cell-intrinsic regulation of the quiescence of naive T cells Nat Immunol, 12(6)(2011), pp. 544-550.

10. H Xiang, W Xue, X Wu, et al. FOXP1 inhibits high glucose-induced ECM accumulation and oxidative stress in mesangial cells Chem Biol Interact, 3132019), pp. 108818.

11. M Yang, H Gan, Q Shen, et al. Proinflammatory CD14+CD16+ monocytes are associated with microinflammation in patients with type 2 diabetes mellitus and diabetic nephropathy uremia Inflammation, 35(1)(2012), pp. 388-396.

12. Y Ikezumi, RC Atkins, DJ Nikolic-Paterson. Interferon-γ augments acute macrophage-mediated renal injury via a glucocorticoid-sensitive mechanism J Am Soc Nephrol, 14(4)(2003), pp. 888-898.

13. T Wada, K Furuichi, N Sakai, et al. Up-regulation of monocyte chemoattractant protein-1 in tubulointerstitial lesions of human diabetic nephropathy Kidney Int, 58(4)(2000), pp. 1492-1499.

14. AS Awad, H You, T Gao, et al. Macrophage-derived tumor necrosis factor-α mediates diabetic renal injury Kidney Int, 88(4)(2015), pp. 722-733.

15. AA Elmarakby, JC Sullivan. Relationship between oxidative stress and inflammatory cytokines in diabetic nephropathy Cardiovasc Ther, 30(1)(2012), pp. 49-59.

16. WA Nockher, JE Scherberich. Monocyte cell-surface CD14 expression and soluble CD14 antigen in hemodialysis: evidence for chronic exposure to LPS Kidney Int, 48(5)(1995), pp. 1469-1476.

17. C Zhao, YC Tan, WC Wong, et al. The CD14(+/low)CD16(+) monocyte subset is more susceptible to spontaneous and oxidant-induced apoptosis than the CD14(+)CD16(-) subset Cell Death Dis, 12010), pp. e95.

18. TS Kapellos, L Bonaguro, I Gemund, et al. Human monocyte subsets and phenotypes in major chronic inflammatory diseases Front Immunol, 102019), pp. 2035.

19. N Yoshida, H Yamamoto, T Shinke, et al. Impact of CD14(++)CD16(+) monocytes on plaque vulnerability in diabetic and non-diabetic patients with asymptomatic coronary artery disease: a cross-sectional study Cardiovasc Diabetol, 16(1)(2017), pp. 96.

20. K Kaur, S Vig, R Srivastava, et al. Elevated hepatic miR-22-3p expression impairs gluconeogenesis by silencing the Wnt-responsive transcription factor Tcf7 Diabetes, 64(11)(2015), pp. 3659-3669.

21. P Jha, H Das. KLF2 in regulation of NF-kappaB-mediated immune cell function and inflammation Int J Mol Sci, 18(11)(2017), pp. 2383.

22. Y Zhu, HK Usui, K Sharma. Regulation of transforming growth factor β in diabetic nephropathy: implications for treatment Semin Nephrol, 27(2)(2007), pp. 153-160.

23. AP Sanchez, K Sharma. Transcription factors in the pathogenesis of diabetic nephropathy Expert Rev Mol Med, 112009), pp. e13.

24. A Rivero, C Mora, M Muros, et al. Pathogenic perspectives for the role of inflammation in diabetic nephropathy Clin Sci, 116(6)(2009), pp. 479-492.

25. SB Gurley, S Ghosh, SA Johnson, et al. Inflammation and immunity pathways regulate genetic susceptibility to diabetic nephropathy Diabetes, 67(10)(2018), pp. 2096-2106.

26. H Yaribeygi, SA-O Atkin, LE Simental-Mendía, et al. Anti-inflammatory effects of resolvins in diabetic nephropathy: mechanistic pathways J Cell Physiol, (1097-4652 (Electronic))(2019).

27. S Mezzano, A Droguett, ME Burgos, et al. Renin-angiotensin system activation and interstitial inflammation in human diabetic nephropathy Kidney Int, 642003), pp. S64-S70.

28. PC Wilson, H Wu, Y Kirita, et al. The single-cell transcriptomic landscape of early human diabetic nephropathy P Natl Acad Sci, 116(39)(2019), pp. 19619-19625.

29. T Li, Z Yu, Z Qu, et al. Decreased number of CD19+CD24hiCD38hi regulatory B cells in Diabetic nephropathy Mol Immunol, 1122019), pp. 233-239.

30. IBM Kolseth, TM Reine, K Parker, et al. Increased levels of inflammatory mediators and proinflammatory monocytes in patients with type I diabetes mellitus and nephropathy J Diabetes Complications, 31(1)(2017), pp. 245-252.

31. E Galkina, K Ley. Leukocyte recruitment and vascular injury in diabetic nephropathy J Am Soc Nephrol, 17(2)(2006), pp. 368-377.

32. JY Moon, KH Jeong, TW Lee, et al. Aberrant recruitment and activation of T cells in diabetic nephropathy Am J Nephrol, 35(2)(2012), pp. 164-174.

33. JW Yoon, S Gollapudi, MV Pahl, et al. Naïve and central memory T-cell lymphopenia in end-stage renal disease Kidney Int, 70(2)(2006), pp. 371-376.

34. X Feng, GC Ippolito, L Tian, et al. Foxp1 is an essential transcriptional regulator for the generation of quiescent naive T cells during thymocyte development Blood, 115(3)(2010), pp. 510-518.

35. FC Brosius, CW Heilig. Glucose transporters in diabetic nephropathy Pediatr Nephrol, 20(4)(2005), pp. 447-451.

36. M Yukawa, S Jagannathan, S Vallabh, et al. AP-1 activity induced by co-stimulation is required for chromatin opening during T cell activation J Exp Med, 217(1)(2020), pp. e20182009.

37. H Yang, T Xie, D Li, et al. Tim-3 aggravates podocyte injury in diabetic nephropathy by promoting macrophage activation via the NF-κB/TNF-α pathway Mol Metab, 232019), pp. 24-36.

38. Y Toda, J Tsukada, M Misago, et al. Autocrine induction of the human pro-IL-1β gene promoter by IL-1β in monocytes J Immunol, 168(4)(2002), pp. 1984-1991.

39. Y Lin, Z Duan, F Xu, et al. Construction and analysis of the transcription factor-microRNA co-regulatory network response to Mycobacterium tuberculosis: a view from the blood Am J Transl Res, 9(4)(2017), pp. 1962-1976.

40. JF Navarro-González, C Mora-Fernández. The role of inflammatory cytokines in diabetic nephropathy J Am Soc Nephrol, 19(3)(2008), pp. 433-442.

41. J Wada, H Makino. Inflammation and the pathogenesis of diabetic nephropathy Clin Sci, 124(3)(2012), pp. 139-152.

42. A Chatzigeorgiou, C Harokopos V Fau - Mylona-Karagianni, E Mylona-Karagianni C Fau - Tsouvalas, et al. The pattern of inflammatory/anti-inflammatory cytokines and chemokines in type 1 diabetic patients over time Ann Med, 42(1365-2060 (Electronic))(2010), pp. 426-438.

43. S Swaminathan, SV Shah. Novel inflammatory mechanisms of accelerated atherosclerosis in kidney disease Kidney Int, 80(5)(2011), pp. 453-463.

44. Q Zhuang, K Cheng, Y Ming. CX3CL1/CX3CR1 axis, as the therapeutic potential in renal diseases: friend or foe? Curr Gene Ther, 17(1875-5631 (Electronic))(2017), pp. 442-452.

45. T-T Chang, J-W Chen. The role of chemokines and chemokine receptors in diabetic nephropathy Int J Mol Sci, 21(9)(2020), pp. 3172.

46. MA Niewczas, ME Pavkov, J Skupien, et al. A signature of circulating inflammatory proteins and development of end-stage renal disease in diabetes Nat Med, 25(5)(2019), pp. 805-813.

47. M Murakoshi, T Gohda, Y Suzuki. Circulating tumor necrosis factor receptors: a potential biomarker for the progression of diabetic kidney disease Int J Mol Sci, 21(6)(2020), pp. 1957.

48. C Lorz, AC Benito A Fau - Ucero, B Ucero Ac Fau - Santamaría, et al. Trail and kidney disease Frontiers In Bioscience, 14(2768-6698 (Electronic))(2009), pp. 3740-3749.

49. C Lorz, A Benito-Martín, A Boucherot, et al. The death ligand TRAIL in diabetic nephropathy J Am Soc Nephrol, 19(5)(2008), pp. 904-914.

**Supplementary Figures**


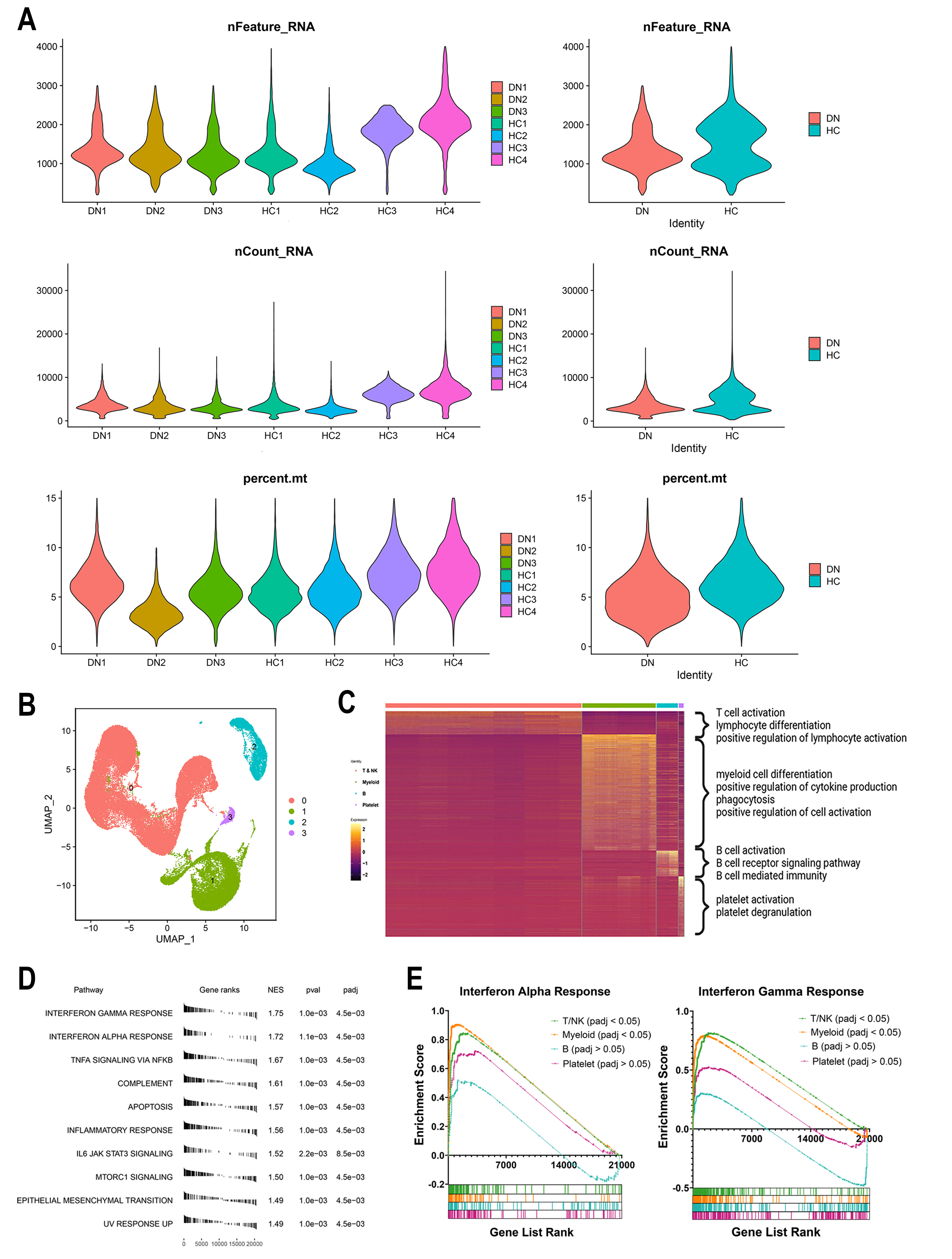


**Figure S1.** Single-cell analysis of four major immune cell types. (**A**) Distribution of nFeature_RNA (number of genes for each cell), nCount_RNA (number of transcripts for each cell) and percent.mt (percentage of mitochondrial genes for each cell) for every sample (left) and two groups (right). (**B**) UMAP plot for four cell types. (**C**) The heatmap showing the expression of all DEGs and specific pathways for four major immune cell types. (**D**) Significantly up-regulated pathways in the DN group. (**E**) Interferon alpha/gamma response for four immune cell types.


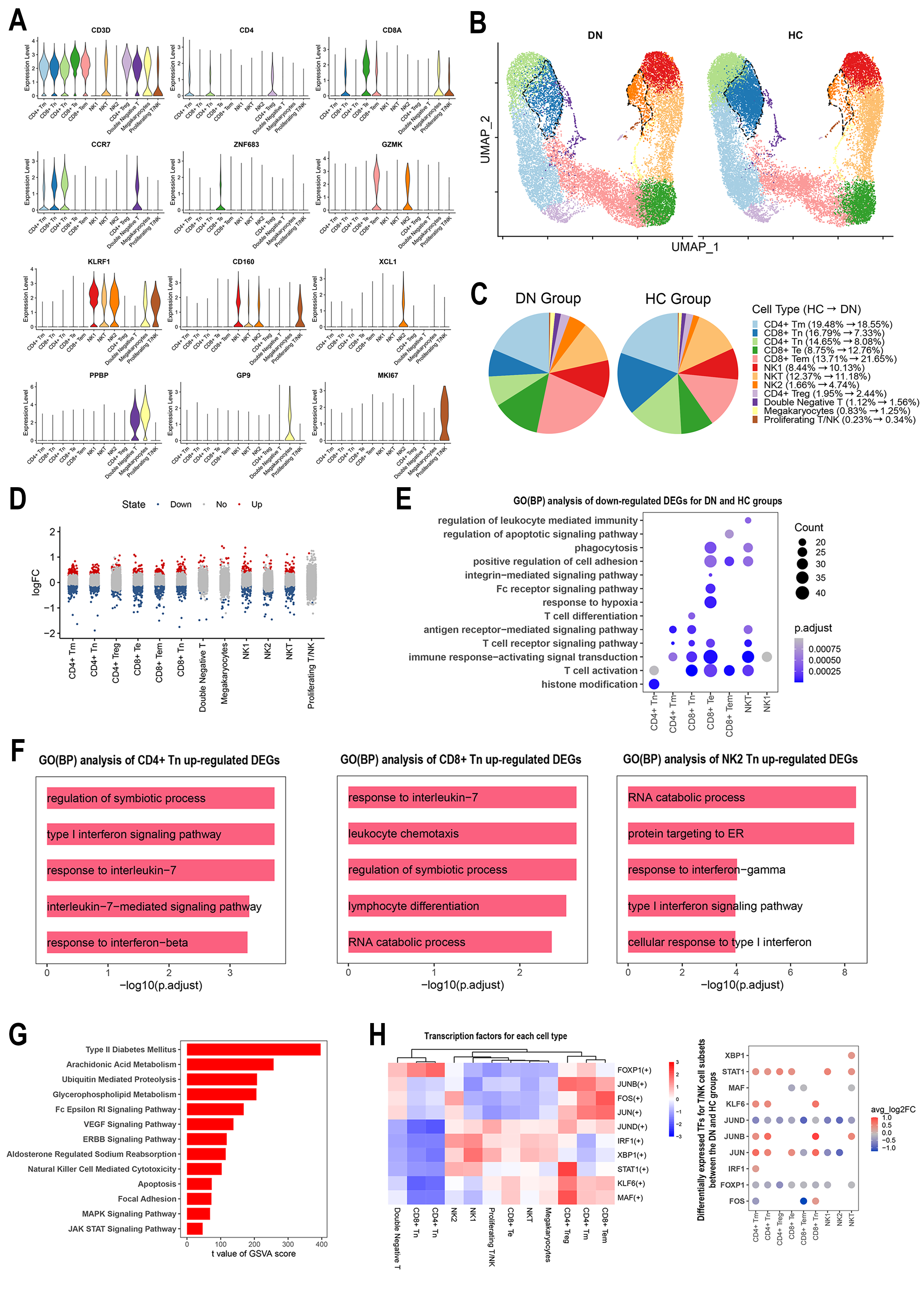


**Figure S2.** Specific changes of T/NK cell subsets between the DN and HC groups. (**A**) The expression of known marker genes for T/NK cell subtypes. (**B**) UMAP plot for T/NK cell subtypes in the two groups. (**C**) Pie plots showing proportion changes of each cell type for two groups. (**D**) DEGs of each cell type. (**E**) GO analysis for up-regulated DEGs of three specific cell types. (**F**) Significantly up-regulated pathways in the DN group. (**G**) The up-regulated pathway activities expressed by t value of GSVA score in DN group. (**H**) Heatmap of the regulon activities score for differentially expressed TFs of T/NK cell subsets (left). Differentially expressed TFs for T/NK cell subsets between the DN and HC groups (right).


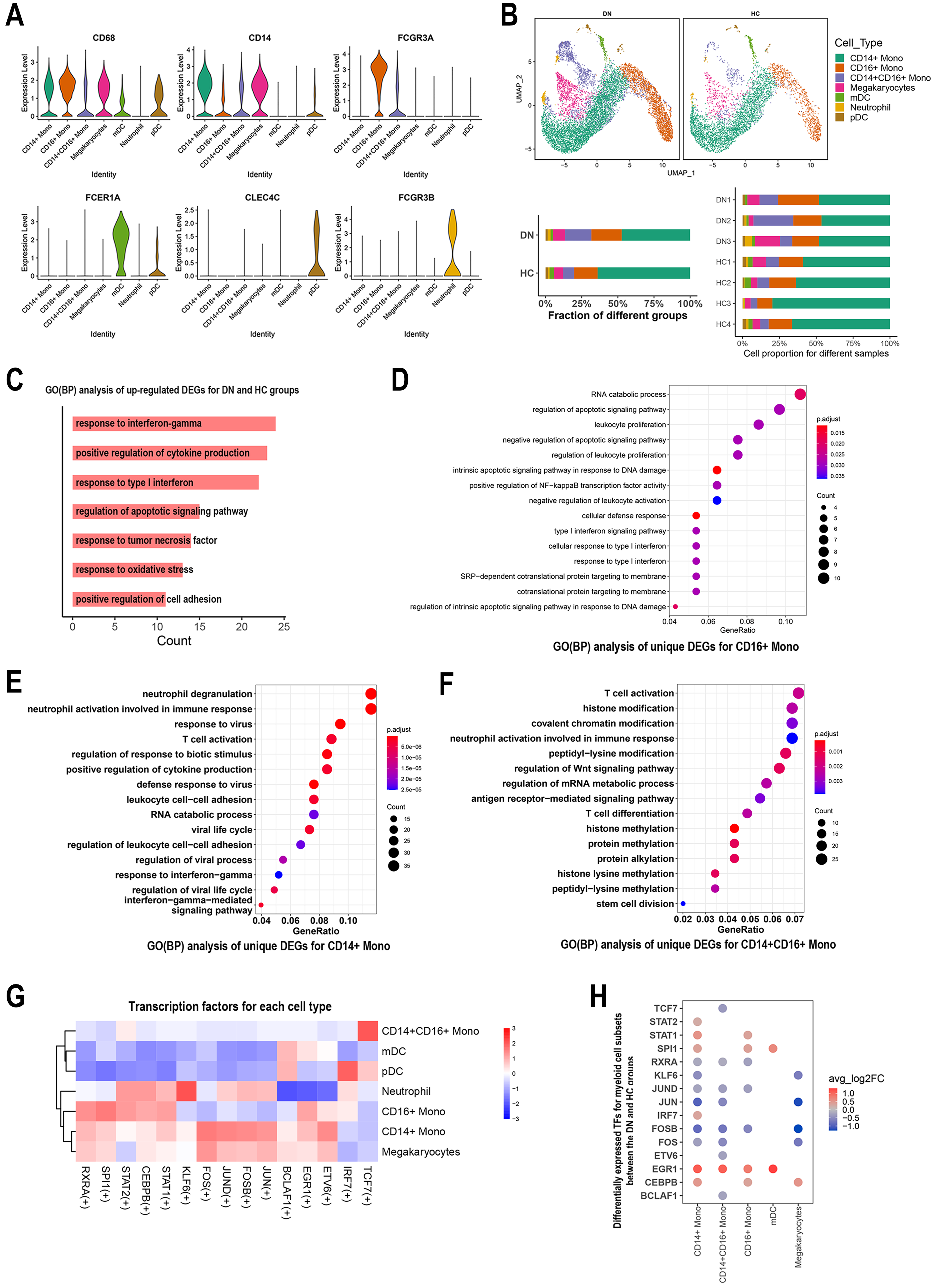


**Figure S3.** Specific changes of myeloid cell subsets between the DN and HC groups. (**A**) The expression of known marker genes for myeloid cell subtypes. (**B**) UMAP plot for myeloid cell subtypes in the two groups (top). Histogram showing proportion changes of each cell type for two groups and samples (bottom). (**C**) GO analysis of up-regulated DEGs for DN and HC groups. (**D**) GO analysis for unique DEGs of CD16+ Mono. (**E**) GO analysis for unique DEGs of CD14+ Mono. (**F**) GO analysis for unique DEGs of CD14+CD16+ Mono. (**G**) Heatmap of the regulon activities score for differentially expressed TFs of myeloid cell subsets. (**H**) Differentially expressed TFs for myeloid cell subsets between the DN and HC groups.


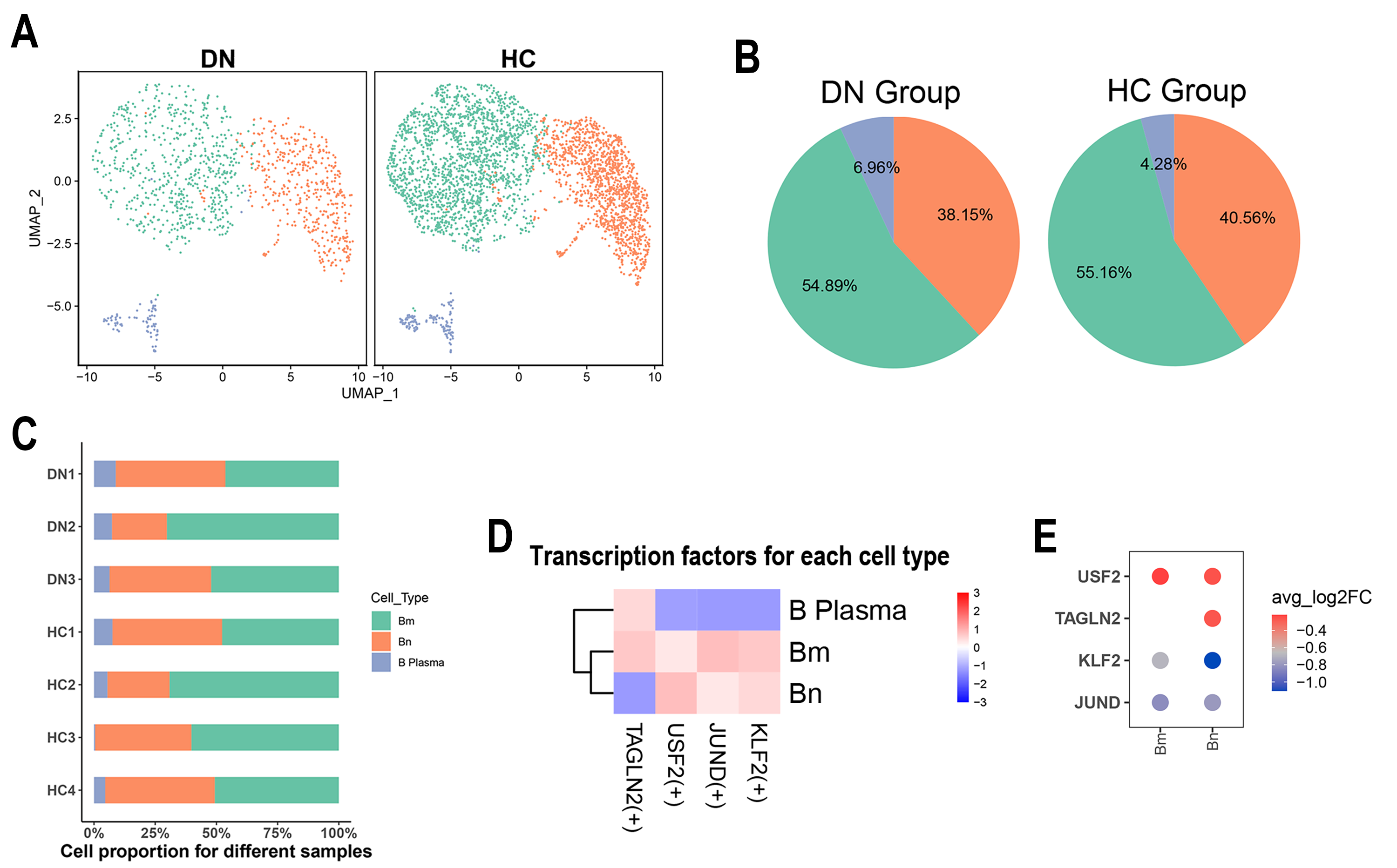


**Figure S4.** Specific changes of B cell subsets between the DN and HC groups. (**A**) UMAP plot for B cell subtypes in two groups. (**B**) Pie plots showing proportion changes of each cell type for two groups. (**C**) Proportion changes of each cell type for every sample. (**D**) Heatmap of the regulon activities score for differentially expressed TFs of B cell subsets. (**E**) Differentially expressed TFs for B cell subsets between the DN and HC groups.


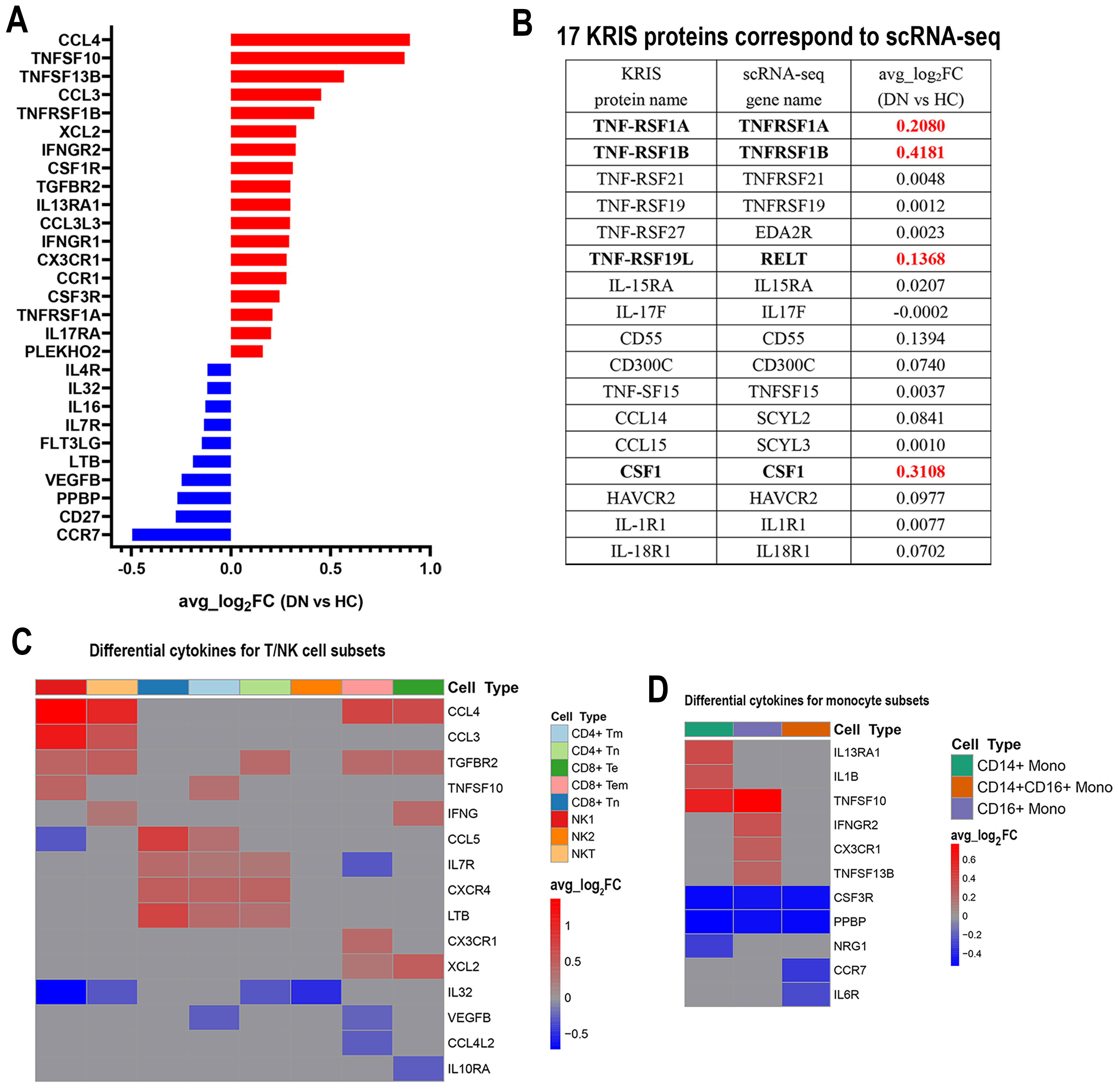


**Figure S5.** Differentially expressed cytokines for two groups and specific cell subtypes. (**A**) Differentially expressed cytokines for the DN and HC groups. (**B**) 17 KRIS proteins correspond to scRNA-seq genes. (**C**) Differentially expressed cytokines for T/NK cell subsets between the DN and HC groups. (**D**) Differentially expressed cytokines for monocyte cell subsets between the DN and HC groups. The red represented high expression and the blue represented the opposite.


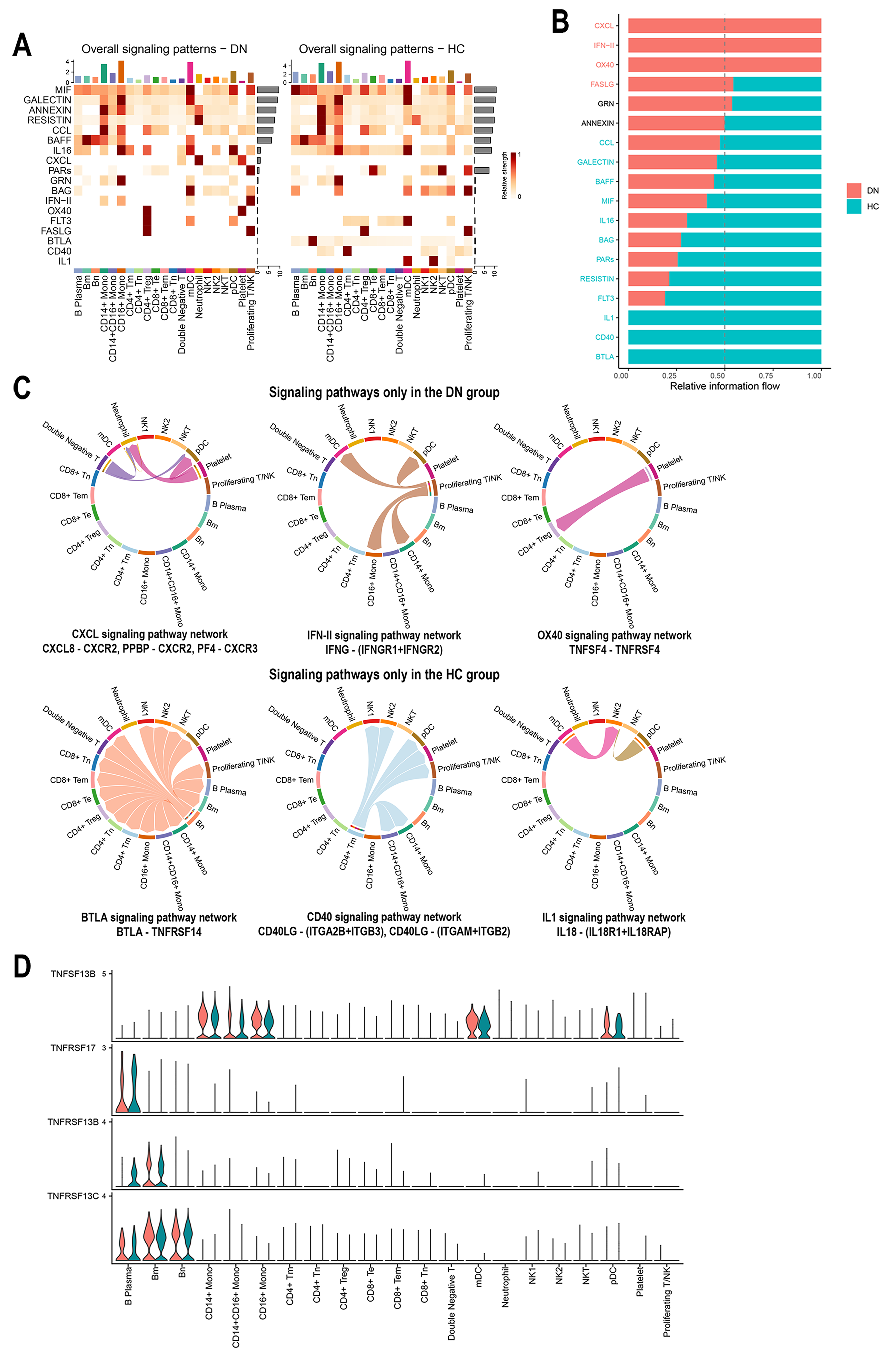


**Figure S6.** Cell-cell communication analysis. (**A**) The overall signaling patterns for the DN and HC groups. (**B**) The relative information flow between the two groups. (**C**) Signaling pathways only in the DN group (top). Signaling pathways only in the HC group (bottom). (**D**) The expression of ligand-receptor genes in the BAFF signaling.

**
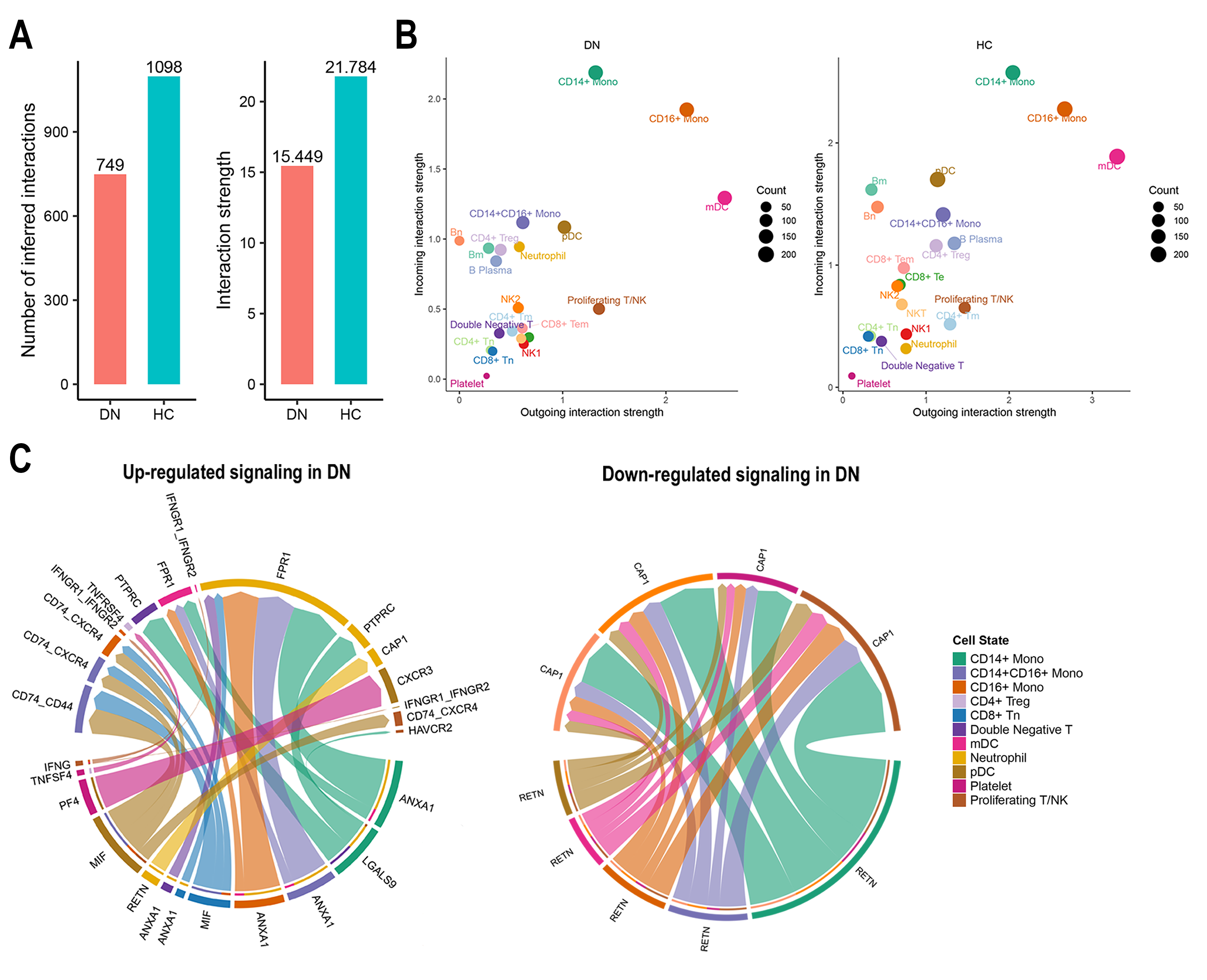
Figure S7.** The information for cell-cell communication analysis. (**A**) The number of inferred interactions and interaction strength for DN and HC groups. (**B**) The interaction strength of every cell type for DN and HC groups. (**C**) Up-regulated and down-regulated signaling in DN.
